# Characteristics of neurological Wilson’s disease with corpus callosum abnormalities

**DOI:** 10.1186/s12883-019-1313-7

**Published:** 2019-05-03

**Authors:** Zhi-Hua Zhou, Yun-Fan Wu, Jin Cao, Ji-Yuan Hu, Yong-Zhu Han, Ming-Fan Hong, Gong-Qiang Wang, Shu-Hu Liu, Xue-Min Wang

**Affiliations:** 1 Department of Neurology, The first affiliated hospital, school of clinical Medicine of Guangdong Pharmaceutical University, Guangzhou, Guangdong China; 2 The second school of clinical Medicine, Southern Medical University, Guangdong Second Provinical General Hospital, Guangzhou, Guangdong China; 3 Department of Orthopaedic, Ningbo No.6 hospital, NingBo, Zhejiang China; 4 Wilson Disease Centre, Hospital Affiliated to Institute of Neurology, Anhui University of Chinese Traditional Medicine, Hefei, Anhui China; 5 Department of Neurobiology, School of Basic Medical Sciences, Southern Medical University, Guangzhou, Guangdong China

**Keywords:** Wilson’s disease, Unified Wilson’s disease rating scale (UWDRS), Magnetic resonance imaging (MRI), Corpus callosum, Neurological dysfunction

## Abstract

**Background:**

Wilson’s disease (WD) is an autosomal recessive disease of impaired copper metabolism. Previous study demonstrated that WD with corpus callosum abnormalities (WD-CCA) was limited to the posterior part (splenium). This study aimed to compare clinical features between WD-CCA and WD without corpus callosum abnormalities (WD-no-CCA).

**Methods:**

Forty-one WD patients who had markedly neurological dysfunctions were included in this study. We retrospectively reviewed clinical, biochemical characteristics and MRI findings in the 41 WD patients. All patients were assessed using the Unified Wilson’s Disease Rating Scale.

**Results:**

Nine patients had corpus callosum abnormalities, 4 of 9 patients had abnormal signal in the genu and splenium, 5 of 9 patients had abnormal signal only in the splenium. WD-CCA had longer course (9.9 ± 4.0 years vs. 3.4 ± 3.6 years, *p*<0.01), more severe neurological dysfunctions (37.6 vs. 65.9, *p*<0.01) and higher psychiatric symptoms scores (11.2 vs. 22.5, *p*<0.01) than WD-no-CCA. The MRI findings indicated that WD-CCA had higher ratio than WD-no-CCA in globus pallidus (88.9% vs. 43.8%, *p* = 0.024) and thalamus (100% vs. 59.4%, *p* = 0.038). The index of liver function and copper metabolism had no significant in WD-CCA and WD-no-CCA patients.

**Conclusion:**

Our findings indicate Wilson’s disease can involve the posterior as well as the anterior part of CC and patients with CC involvement had more extensive brain lesions, more severe neurological dysfunctions and psychiatric symptoms.

## Background

Wilson’s disease (WD), also known as hepatolenticular degeneration, was first described by the British neurologist Kinnier Wilson in 1912. WD is an autosomal recessive disease of impaired copper metabolism [1].The causative gene of WD is ATP7B, which encodes a copper-transporting ATPase in the liver and functions as a copper-dependent P-type ATPase [2]. Clinical manifestations of WD include neurological, liver, renal, and psychosis symptoms, as well as Kayser-Fleischer rings (K-F rings) of the cornea [3]. Brain magnetic resonance imaging (MRI) in WD has demonstrated a significant correlation with clinical findings, and interval changes on follow up MRI were also closely correlated with clinical findings, which have been helpful in assessing clinical response [4, 5].

Abnormal corpus callosum (CC) in WD have rarely been addressed and limited to the posterior part (splenium) in previous studies [6]. In China, the study of WD has made great progress [7]. During the past 2 years, we have collected nine Chinese WD patients presenting with corpus callosum abnormalities (WD-CCA) at the Wilson’s Disease Centre, Hospital Affiliated with the Institute of Neurology, Anhui University of Chinese Traditional Medicine. The aim of this study is to evaluate the frequency of CC lesions involvement in patients of WD; and to study differences in the clinical, biochemical, and neuroimaging features in patients with or without CC involvement.

## Methods

Forty-one Wilson’s disease patients who exhibited prominent neurological dysfunction were retrospectively analyzed in this study from July 2014 to June 2016 at Wilson’s Disease Centre Hospital Affiliated to Institute of Neurology, Anhui University of Traditional Chinese Medicine. All patients met the diagnostic criteria for WD [8]. All MRI scans were performed on a 1.5-T Philips Achieva MRI scanner with a 32-channel SENSE head coil. All the patients were performed the following sequences: T1- and T2- weighted scans, fluid attenuated inversion recovery (FLAIR). The presence of lesions on MRI in the following structures was collected: basal ganglia, cerebellum, midbrain, corpus callosum (CC) and other localisations in white matter. All patients’ symptom severity with respect to neurological, liver and psychosis symptoms were evaluated using the Unified Wilson’s Disease Rating Scale (UWDRS) for WD [9, 10]. Demographic and clinical characteristics of the 41 WD patients are shown in Table 1.

**Table 1:** Clinical and biochemical characteristics of Wilson’s disease patients with corpus callosum abnormalities and without corpus callosum abnormalities

	WD-no-CCA	WD-CCA	*P* value
Gender(male/female)	23/9	6/3	1.00
Age of onset	17.4 ± 6.1 years	17.8 ± 3.8 years	0.87
From onset to corpus callosum abnormal	–	9.2 ± 4.8 years	–
Course of the disease	3.4 ± 3.6 years	9.9 ± 4.0 years	0.00
UWDRS Neurological symptoms score	37.6 (35–38)	65.9 (64–68)	0.00
UWDRS Hepatic symptoms score	5.7 (2–12)	5.8 (3–11)	0.47
UWDRS Psychiatric symptoms score	11.2 (9–14)	22.5 (20–25)	0.00
Total bilirubin (μmol/L)	14.6 ± 6.3	12.0 ± 5.0	0.26
Direct bilirubin (μmol/L)	4.0 ± 1.9	4.6 ± 2.7	0.44
Serum albumin (g/L)	43.6 ± 4.3	43.4 ± 2.8	0.89
Serum globulin (g/L)	25.0 ± 4.4	27.0 ± 5.4	0.26
Alanine aminotransferase (U/L)	27.5 ± 10.7	22.3 ± 7.2	0.45
Aspartate aminotransferase (U/L)	28.9 ± 14.3	21.6 ± 9.9	0.16
24-h urinary copper (μg)	213.0 ± 167.2	296.0 ± 115.1	0.18
Serum ceruloplasmin (μg/mL)	73.1 ± 40.8	49.0 ± 13.7	0.09
Serum copper (μmol/L)	3.5 ± 2.5	3.7 ± 1.2	0.76
K-F ring	32 (100%)	9 (100%)	–

Neurological, hepatic and psychiatric symptoms score of UWDRS were presented by median and 25–75% confidence interval were presented in bracket. *WD-no-CCA* WD without corpus callosum abnormalities, *WD-CCA* WD with corpus callosum abnormalities; Values in parenthesis indicate percentage

Venous blood was used to detect levels of total bilirubin, direct bilirubin, albumin, globulin, alanine aminotransferase, aspartate aminotransferase, ceruloplasmin, and copper in 41 WD patients. We also detected levels of 24-h urinary copper in WD patients. Serum ceruloplasmin (Cp) was detected by immunoturbidimetry methods in an automatic biochemical analyzer (Hitachi7180). Serum and 24-h urinary copper were assessed by atomic absorption spectroscopy.

Statistical analysis was performed using SPSS version 19.0 for Windows (SPSS IBM; Chicago, IL, USA). An independent-samples T test analysis of variance test was used to compare age of onset, course of the disease, total bilirubin, direct bilirubin, serum albumin, serum globulin, alanine aminotransferase, aspartate aminotransferase, 24-h urinary copper, Cp and serum copper. A Mann-Whitney U-test was used to compare symptoms score of neurological, hepatic and psychiatric of UWDRS. A Pearson Chi-squared test or Fisher’s exact test was used to compare sex and K-F ring test results. A p<0.05 considered to be statistically significant.

## Results

### Differences in the clinical features of WD with or without CC involvement

All 41 patients denied WD family history and had Kayser-Fleischer rings in the cornea. Diagnostic scores [8] of 9 WD-CCA were greater than or equal to 7 points, and another 32 WD-no-CCA were greater than or equal to 5 points. In WD-CCA patients, 4 patients exhibited signal changes in the genu and splenium corpus callosi, and another 5 patients only exhibited signal changes in the splenium corpus callosum.

As shown in Table 1, gender and age of onset were not significantly different between WD-CCA and WD-no-CCA. However, WD-CCA patients had a longer disease course than WD-no-CCA patients (9.9 ± 4.0 years vs. 3.4 ± 3.6 years, *p* = 0.00). In addition, the time from onset to discovery of corpus callosum abnormalities was 9.2 ± 4.8 years.

We assessed the severity of patients’ conditions using the UWDRS [9, 10]. The UWDRS contains three items, including neurological function, psychiatric symptoms, and hepatic clinical signs. As shown in Table 1, WD-CCA patients exhibited higher scores than WD-no-CCA patients in neurological function (65.9 ± 1.8 vs. 36.4 ± 6.7, p = 0.00) and psychiatric symptoms (22.2 ± 2.7 vs. 10.3 ± 3.1, p = 0.00). Hepatic symptom scores were not significantly different between WD-CCA and WD-no-CCA patients.

In summary, WD-CCA patients have a longer disease course and more severe neurological dysfunction than those without corpus callosum abnormalities.

### Biochemical characteristics in WD with or without CC involvement

As shown in Table 1, none of these results were significantly different between WD-CCA and WD-no-CCA patients.

In summary, the index of liver function and copper metabolism are not significantly different between WD-CCA and WD-no-CCA patients.

### Differences in the neuroimaging features of patients with or without CC involvement

**Fig. 1:**
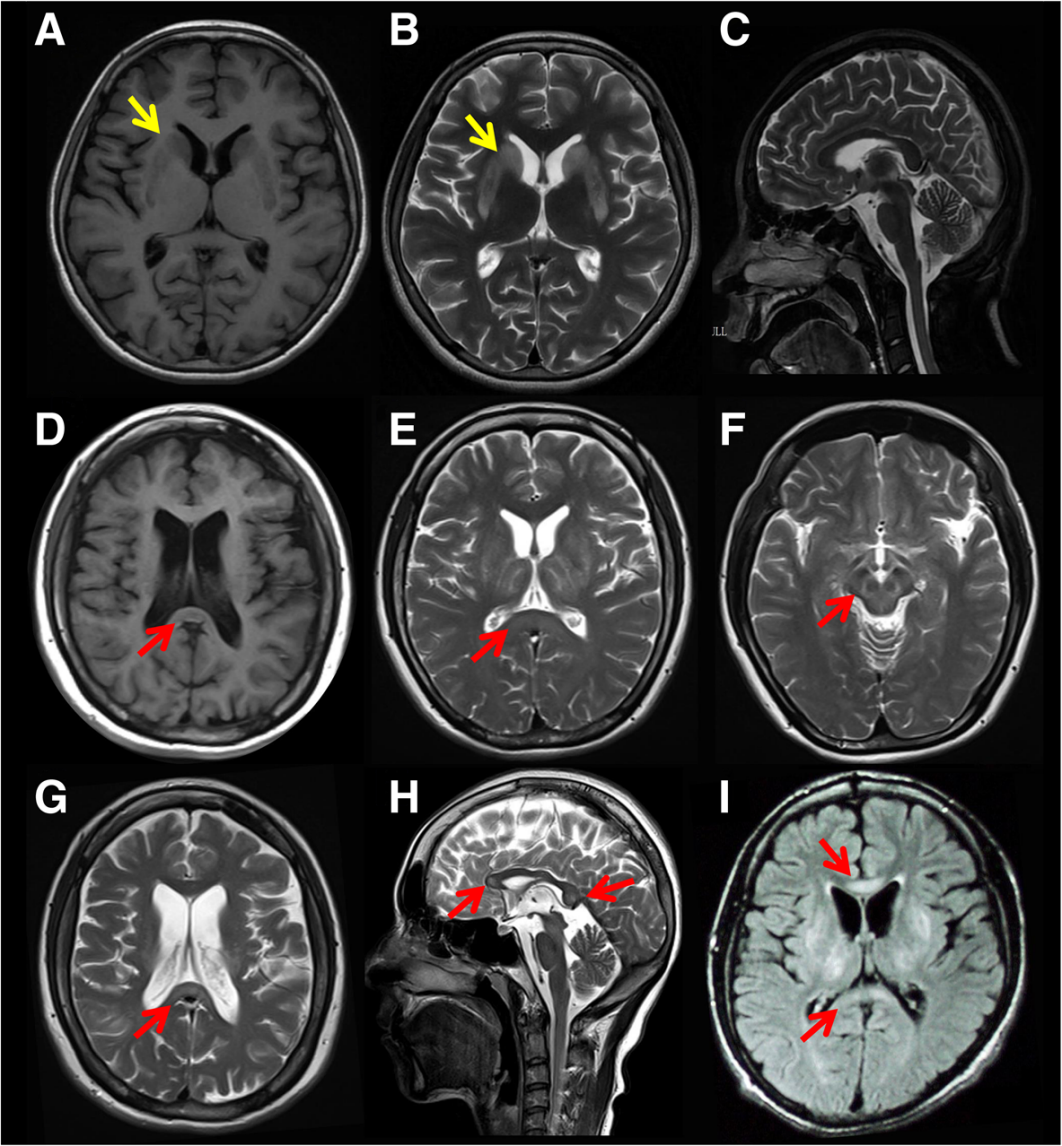
Representative MR image of WD with (WD-CCA) and without corpus callosum abnormalities (WD-no-CCA). **a**-**c** Representative MR image of WD without corpus callosum abnormalities. **d**-**i** Representative MR image of WD without corpus callosum abnormalities. **a**-**c** Bilateral, symmetrical abnormal signals of caudate nucleus and putamen in T1- and T2-weighted axial sequence MRI (yellow arrow). **d** T1-weighted axial MRI demonstrates that bilateral anterior horn of lateral ventricle enlarged and hypointensities in Splenium. **e** T2-weighted axial MRI demonstrates: bilateral, symmetrical hyperintensities in lenticular nucleus, thalamus and hyperintensity in Splenium. **f** T2-weighted axial MRI demonstrates face of the giant panda in brain stem, **g** T2-weighted axial MRI demonstrates hyperintensities in Splenium. **h** T2-weighted sagittal MRI demonstrates hyperintensities in the genu and splenium. **i** Fluid-attenuated inversion recovery sequence, axial MRI demonstrates hyperintensities in genu and splenium, and bilateral, symmetrical hyperintensities in putamen, globus pallidus, caudate nucleus and thalamus

In 41 patients, 9 WD patients had corpus callosum abnormalities and another 32 WD patients who did not present with corpus callosum abnormalities as assessed by magnetic resonance imaging (MRI). As shown in Table 2, in WD-CCA patients, MRI revealed signal changes in the putamen (100%), globus pallidus (88.9%), caudate nucleus (55.6%), thalamus (100%), and brain stem (66.7%), as well as ventricular widening (77.8%). In WD-no-CCA patients, MRI revealed signal changes in the putamen (100%), globus pallidus (43.8%), caudate nucleus (68.8%), thalamus (59.4%), and brain stem (40.6%), along with ventricular widening (77.8%). WD-CCA patients have higher a ratio of changes in the globus pallidus (88.9% vs. 43.8%, *p* = 0.024) and thalamus (100% vs. 59.4%, *p* = 0.038) on MRI than WD-no-CCA patients. However, there was no significant difference between the putamen, globus pallidus, brain stem, or ventricular widening between these two patient groups. A representative MR image is shown in Fig. 1. In summary, MRI findings revealed that WD-CCA patients have more extensive brain lesions than WD-no-CCA patients.

**Table 2:** MRI findings of 9 Wilson’s disease patients with corpus callosum abnormalities compared to without corpus callosum abnormalities

	WD-no-CCA	WD-CCA	*P* value
Putamen (lesions)	32 (100%)	9 (100%)	–
Globus pallidus (lesions)	14 (43.8%)	8 (88.9%)	0.024
Caudate nucleus (lesions)	22 (68.8%)	5 (55.6%)	0.692
Thalamus (lesions)	19 (59.4%)	9 (100%)	0.038
Ventricular widening	13 (40.6%)	7 (77.8%)	0.067
Brain stem (lesions	14 (43.8%)	6 (66.7%)	0.277
Genu corporis callosi	–	4 (44.4%)	–
Splenium corporis callosi	–	9 (100%)	–

*WD-no-CCA* WD without corpus callosum abnormalities, *WD-CCA* WD with corpus callosum abnormalities; Values in parenthesis indicate percentage

## Discussion

In 2010, Trocello and colleagues reported that WD-CCA is not unusual (23.4%) and that corpus callosum signal changes should suggest a diagnosis of WD [6]. However, all corpus callosum signal changes were limited to the posterior (splenium) [6]. We retrospectively reviewed clinical and biochemical characteristics, along with MRI findings, of 9 WD Chinese patients with corpus callosum abnormalities. Our results indicate that WD-CCA is not limited to the posterior (splenium) and that WD-CCA exhibited a longer course of disease, more severe neurological dysfunction, and more extensive brain lesions compared to WD-no-CCA patients.

Along with advances in MRI technology, some new technologies are being used to diagnose and evaluate WD [11–15]. The most frequent findings are increased density on computerized tomography or hyperintensity on T2 MRI in the basal ganglia [3]. In recent years, some unusual MRI findings have been reported, such as face of the giant panda [16–18], eye of tiger [19], central pontine myelinolysis (CPM)-like signal changes [20], and so on. Therefore, MRI is an important and useful tool in WD. In our study, we found that 9 of 41 WD patients exhibited corpus callosum abnormalities (21.95%): 4 patients presented with signal changes in the genu and splenium corpus callosi, which are not limited to the posterior (splenium) [6], and another 5 patients only showed signal changes in the splenium corpus callosum. This ratio is almost the same as in Trocello’s report [6]. As far as we know, in addition to Trocello’s report [6], no other reports have specifically evaluated WD with corpus callosum abnormalities. Hence, we cannot conclude that corpus callosum abnormalities are particularly unusual in WD. Our study demonstrates that these patients exhibit more extensive brain lesions than WD-no-CCA patients. WD-CCA patients have a higher ratio of signal changes in the globus pallidus and thalamus by MRI than WD-no-CCA patients.

We also assessed the neurological functions, hepatic symptoms, and psychiatric symptoms using the UWDRS [6, 9]. The UWDRS is a promising tool to assess disease severity in WD [10]. Our results indicated that WD-CCA patients have more severe neurological dysfunction and psychiatric symptoms than WD-no-CCA patients. Hepatic symptom scores for the UWDRS were not significantly different between WD-CCA and WD-no-CCA patients. This result is likely related to the controls, for which all patients exhibited prominent neurological dysfunction and no prominent hepatic symptoms. There was no significant difference between WD-CCA and WD-no-CCA patients in detected biochemical characteristics. In addition, WD-CCA patients have a longer disease course than WD-no-CCA patients. Therefore, we speculate that more severe neurological dysfunction and psychiatric symptoms in WD-CCA patients may be related to the longer disease course.

## Conclusions

In summary, our retrospective study demonstrated that CC involvement (both anterior and posterior) can occur in WD, the radiologist and clinicians should keep this minds. WD-CCA patient exhibit more extensive brain lesions, along with more severe neurological dysfunction and psychiatric symptoms. We speculate that WD-CCA patients who present with more severe neurological dysfunction and psychiatric symptoms may due to longer disease course. Because of the small sample size of this study, we cannot conclude that corpus callosum abnormalities are not unusual in WD [6]. We should further study with larger sample size and may convey more information regarding the frequency and significance of CC involvement in WD patients.

## Abbreviation

Cp: Ceruloplasmin.
MRI: Magnetic resonance imaging
UWDRS: Unified Wilson’s Disease Rating Scale
WD: Wilson’s disease
WD-CCA: WD with corpus callosum abnormalities
WD-no-CCA: WD without corpus callosum abnormalities
